# Breast cancer and occupation: Non-parametric and parametric net survival analyses among Swiss women (1990–2014)

**DOI:** 10.3389/fpubh.2023.1129708

**Published:** 2023-04-05

**Authors:** Irina Guseva Canu, Nicolas Bovio, Patrick Arveux, Jean-Luc Bulliard, Evelyne Fournier, Simon Germann, Isabelle Konzelmann, Manuela Maspoli, Elisabetta Rapiti, Michel Grzebyk

**Affiliations:** ^1^Center for Primary Care and Public Health (Unisanté), University of Lausanne, Lausanne, Switzerland; ^2^Neuchâtel and Jura Cancer Registry, Neuchâtel, Switzerland; ^3^Geneva Cancer Registry, University of Geneva, Geneva, Switzerland; ^4^Valais Cancer Registry, Valais Health Observatory, Sion, Switzerland; ^5^Department of Occupational Epidemiology, National Research and Safety Institute (INRS), Vandoeuvre lès Nancy, France

**Keywords:** occupational epidemiology, cohort, cancer registry, breast cancer, female worker, return to work, occupational exposure

## Abstract

**Introduction:**

Occupation can contribute to differences in risk and stage at diagnosis of breast cancer. This study aimed at determining whether occupation, along with skill level and the socio-professional category, affect the breast cancer survival (BCS) up to 10 years after diagnosis.

**Materials and methods:**

We used cancer registry records to identify women diagnosed with primary invasive breast cancer in western Switzerland over the period 1990–2014 and matched them with the Swiss National Cohort. The effect of work-related variables on BCS was assessed using non-parametric and parametric net survival methods.

**Results:**

Study sample included 8,678 women. In the non-parametric analysis, we observed a statistically significant effect of all work-related variables on BCS. Women in elementary occupations, with low skill level, and in paid employment not classified elsewhere, had the lowest BCS, while professionals, those with the highest skill level and belonging to top management and independent profession category had the highest BCS. The parametric analysis confirmed this pattern. Considering elementary occupations as reference, all occupations but Craft and related trades had a hazard ratio (HR) below 1. Among professionals, technicians and associate professionals, and clerks, the protective effect of occupation was statistically significant and remained unchanged after adjustment for age, calendar period, registry, nationality, and histological type. After adjusting for tumor stage, the HRs increased only slightly, though turned non-significant. The same effect was observed in top management and independent professions and supervisors, low level management and skilled laborers, compared to unskilled employees.

**Conclusion:**

These results suggest that work-related factors may affect BCS. Yet, this study was conducted using a limited set of covariates and a relatively small study sample. Therefore, further larger studies are needed for more detailed analyses of at risk occupations and working conditions and assessing the potential interaction between work-related variables and tumor stage.

## 1. Introduction

### 1.1. Epidemiology of breast cancer

Breast cancer (BC) is the most frequent cancer in women, representing 25% of malignancies (1). In Switzerland, BC is considered a public health priority; with 31% of new cancer cases, it accounts for the highest number of potential life-years lost before age 70 (2). Since 2003–2007, BC incidence started to decline in most high income countries, including Switzerland (3). Nevertheless, in younger women, an increasing trend was recently reported in Switzerland and some other European countries (4–7).

With screening generalization and the progress made in treatment, the mortality has been constantly declining and survival improving. Regarding the BC prognosis, Switzerland ranks the best among the European countries (8). The age-standardized relative cumulative survival at 10 years after BC diagnosis is currently estimated at 77.5% (9). However, depending on tumor stage at diagnosis, it ranges from 94.5% for Stage I to 9.3% for Stage IV (8).

While tumor stage and age at diagnosis are two major prognostics factors, other factors including tumor characteristics, treatment, comorbidities, and socioeconomic status have been suggested to impact the BC survival (8, 10). Yet, neither socioeconomic differences in stage at diagnosis nor other sociodemographic factors such as age, nationality and marital status could explain the survival inequalities observed in Switzerland (11). In contrast, age beyond of the recommended screening age, unmarried marital status, low socioeconomic status, and residence in a canton without organized BC screening program were associated with an increased risk of being diagnosed with a later-stage BC (11). Moreover, after controlling for calendar time, canton, age, marital status and nationality, BC stage at diagnosis was found gradually associated with the socio-professional category and skill level required for the occupation. This gradient was also observed for occupation and occupational activity sector, though to a lesser extent (12).

### 1.2. Occupation and breast cancer

Occupation is a key variable in occupational health since work is an important health determinant (13, 14). Occupation enables estimating or approximating the working conditions and exposures to carcinogens in the workplace and analyzing their effect on incidence and mortality from specific causes of death, including cancer (15). Occupation influences both incidence and mortality of BC (12). As a result, several occupations have been identified at risk of BC, namely occupations in dry cleaning, hairdressing, metalworking, aircraft maintenance, textile, leather and fur processing, electronics manufacturing, and military, as well as dentists, physicians, journalists, administrators, flight attendants and artistic workers (12, 16–19).

The fraction of BC attributable to occupational exposures to ionizing radiation, shift work and cytostatic drugs was estimated at 9.8% as a central core estimate and 18.5% as a midpoint estimate (20). Yet, women can be exposed to many other risk factors in the workplace (21), namely the circadian rhythm disruptors through night shift work (22–24), electromagnetic fields (25), and carcinogenic metals and chemicals (19, 26). Compared with occupational exposures, the first established risk factors of BC (i.e., family BC history and reproductive, hormonal, and morphological characteristics) account for less than 30% of incident cases (27, 28). A high fasting glucose, high BMI, and diet high in red meat were identified as having the highest estimated fractions of BC deaths and DALYs attributable to metabolic risk factors (6.1%, 4.7%, and 3.2% of DALYs, respectively) (29). Among behavioral risk factors, alcohol consumption ranks the first (5.2% of DALYs), followed by second hand smoke exposure and smoking (2.7% and 2.5%, respectively), and by low physical activities (1.0% of DALYs) (29). Occupation thus plays a critical role in the onset of BC, contributing directly or indirectly to the development and progression of BC (19, 30).

**Figure 1:**
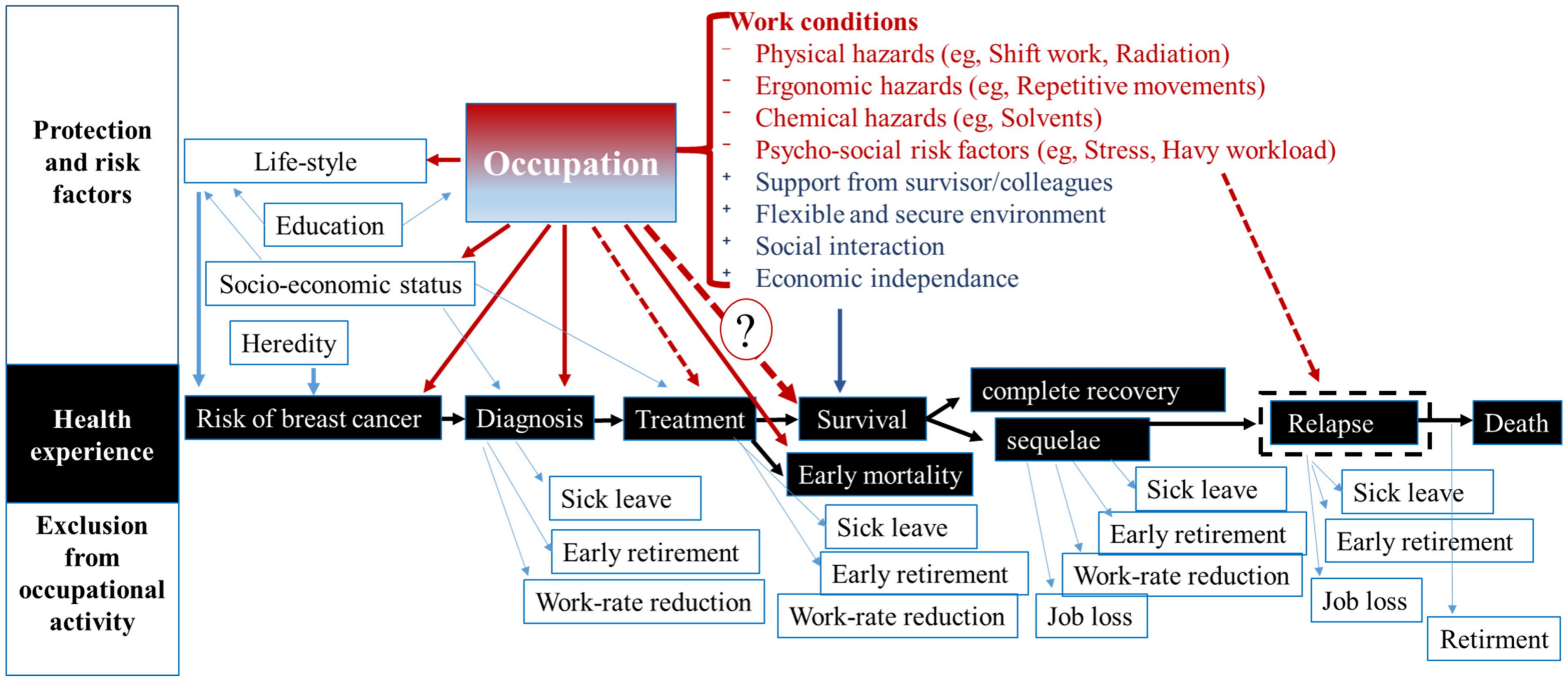
Relationships between occupation and breast cancer experience for a working woman. Dot-lines correspond to the hypothesized relationships, which have not been studied yet. Question mark indicates the relationship investigated in this study. Text in red corresponds to examples of breast cancer risk factors, text in blue corresponds to examples of protective factors.

Occupation and work-related factors can impact the woman’s life-course at virtually all steps of her BC journey (Figure 1). For instance, depending on occupation, flexible working hours, supportive supervisors and availability of occupational health programs and services could facilitate employee’s participation in BC screening and an early BC diagnosis, or conversely, hamper it. In our previous studies, we found an association between occupation and tumor stage at diagnosis for lung and BC (12, 15).

Similarly, BC treatment can be more or less compatible with usual working conditions and influence the treatment efficacy. As some antineoplastic drugs come with side-effects such as ototoxicity, neurotoxicity and nephrotoxicity (31–33), the exposure to noise, solvents, and some metals in the workplace can worsen the treatment tolerance or make it incompatible with work. Exposure to artificial light during night changes the expression of circadian genes acting as tumor suppressors (34, 35). Altered expression of these genes is related with atypical cell proliferation, DNA repair impairment and apoptosis. It also increases human cancer cells’ drug resistance (22, 36). A suboptimal or ineffective treatment is related to a poorer survival and occupation can modify this relationship. Although research has still neglected this topic, an excessive mortality from BC in some occupations supports this hypothesis.

Regarding the relationship between occupation and survival, the evidence comes from studies on return to work (RTW) after BC diagnosis and treatment (37–41). This research shows that for many women, RTW is a “symbol of recovery,” raising their self-esteem and helping overcome the treatment side effects (42–44). Compared with other cancers, BC survivors have the greatest chance of RTW (41). Consequently, work ability, social reintegration and (re-)employment are a highly relevant concern for BC survivors. Most of them (85%) are motivated either to return to work or be re-employed after rehabilitation (45, 46). However, many BC survivors experience a significantly increased risk for unemployment and early retirement (47–49), as shown in Figure 1.

The prevalence of RTW after a treatment–related sick-leave depends on national legislation (45, 50), yet in liberal countries like Switzerland or South Korea, it can be company and occupation dependent. Availability of the rehabilitation and support systems both in hospitals and workplace enabling to “work around the treatment schedule” (40) or gradually resuming work in accordance with their health condition are facilitators of RTW. Indeed, ongoing physical and/or cognitive limitations, such as persisting fatigue, treatment-induced menopausal symptoms, difficulties with lifting, coping issues, anxiety, and depression -common in BC survivors- can limit their workability and require a work rate adjustment (37, 51–54). While reduction of working hours is associated with financial difficulties, these difficulties are more important among those who had to quit their job after RTW (55–57).

The probability of RTW is strongly related with younger age at diagnosis, less advanced stage of BC, higher education, non-manual work, or being self-employed. However, in some countries, self-employed workers, especially those on short-term contracts or employed by very small companies (with less than 5 employees) are less protected by employment lows and have limited right to paid sick-leave. Workplace policies, procedures, culture, but also workload emerged as major factors impacting RTW experience in many studies (37, 39, 43, 58). Thus, cancer survivors able to return to work usually have unstable employment trajectories than other workers (45, 59). As a result, occupation-related and financial factors that could vary while the disease can damage their quality of life and, consequently, their survival (53, 60).

### 1.3. Research question and hypotheses

Occupation has been frequently and relevantly used as a proxy of occupational exposures, either directly or in linkage with job-exposure matrices (61). In line with a life-course exposure model “Exposome” (62), the “Worksome” has been proposed for the “explicit consideration of both physical and psychosocial exposures and effects derived from work and working conditions” (63). The authors of the worksome framework concluded that the relationship between health and work should be examined with classifications specific to occupation or industry instead of socio-economic class classifications (63). Therefore, in this study, we aimed to assess the relationship between occupation and BC survival among women with known occupation living in Western-Switzerland. As BC incidence and stage at diagnosis was found related to the skill level required for occupation and the socio-professional category we hypothesized that these variables can be also associated with BC survival.

## 2. Materials and methods

In this study we applied the methodology developed for our previous study dedicated to the lung cancer survival (15). To help the reader understand it, we summarized below the most important points.

### 2.1. Study sample, predictors, and outcome definition

Data from the cancer registries of Western Switzerland (cantons of Geneva, Neuchâtel, Vaud, and Wallis) for the period 1990–2014 were used as source data. These data were matched with the Swiss National Cohort (SNC) to retrieve information on occupation and mortality. The SNC is based on data from the 1990 and 2000 federal censuses, which were linked to mortality, birth, and emigration records (64). The SNC has an estimated population coverage of 98.6% (65).

The study sample included female breast cancer cases aged between 18 and 65 years at the time of either of the census with a known occupation. Participants were followed from the date of breast cancer diagnosis until the earliest of the following events: date of emigration, 85th birthday, death, or study termination (December 31, 2014).

Three different work-related variables were analyzed as independent predictors. The first was the participant’s occupation, which was collected twice (in 1990 and 2000) and coded according to the International Standard Classification of Occupations, 1988 version (ISCO-88) established by the International Labour Organization (ILO). In this study, we used the first digit of the ISCO-88 code, which identifies nine major occupational groups such as clerks or technicians. The skill level required for the occupation was the second predictor variable. This variable was also established by the ILO (66). Finally, the socio-professional category was the third work-related variable considered. It was defined by the Swiss Federal Statistical Office (SFSO) as a composite of occupation, the highest level of education completed, occupational status, and legal form of business (67). As start and end dates of employment were not known, we assigned the 1990 census occupational information for participants diagnosed with BC between 1990 and 2000, and the 2000 information thereafter.

The study outcome was incident primary malignant BC, coded C50 based on the International Classification of Diseases for Oncology (ICD-O), 3rd edition. BC cases were selected according to the International Agency for Research on Cancer (IARC) rules for multiple primary cancers (68). Cases were grouped according to the histological types into the following categories: ductal carcinoma (8,500, 8,522 and 8,523), lobular carcinoma (8,520 and 8,524) and other, excluding sarcomas (8,800) and lymphomas (9,590) localized in the breast.

Cancer registries coded tumor stage at diagnosis according to the classification of malignant tumors (TNM) (69). Tumors localized to the organ of origin constituted stages I and II, locally extensive spread, particularly to regional lymph nodes, stage III, and tumors with distant metastasis, stage IV. When missing, stage at diagnosis was imputed, using multivariate imputation by chained equations (70). Nelson−Aalen cumulative hazard, cancer registry, language region, age at diagnosis, marital, status skill level required for the occupation, and socio-professional category were used as stage predictors. All models were run with 25 imputations, in order to reduce the impact of the random sampling inherent in multiple imputation procedures (71). Stages III and IV were grouped in one category, since it allowed a better match when comparing the proportions of each stage between the observed and imputed data.

### 2.2. Statistical analyses

Net survival can be used to estimate the survival that would be observed if the only possible underlying cause of death was the disease under study (72). Prior findings showed that the relative survival approach was more robust than the cause-specific one and more suitable for net survival analysis (73). In the relative survival setting, the cause of death of participants who die during follow-up is ignored; net survival is estimated using life tables and can be defined as the ratio of the observed survival to the one expected from the life tables (74). In other words, it approximates the net survival probability and can be seen as the survival probability from the disease under study after all other risk have been removed.

In this study, we applied two methods of net survival analysis. First, the Pohar-Perme non-parametric method (75) was used along with the log-rank type test (76) to compare the net survival curves between groups. Secondly, for every predictor variable, we applied a parametric method that models the excess hazard in a framework of multivariable proportional hazard regression model (77).

For the non-parametric approach, we used the STNS package (78) developed in STATA. It requires the all-cause mortality rate table, which is used to compute the expected hazard and survival of each subject at each event time in the dataset. The latter was calculated using the mortality rates of the female population of the cantons of Geneva, Neuchâtel, Vaud, and Wallis stratified by 5-year age group (18–85 years) and 5-year calendar period (1990–2014). These categories were chosen to smooth the rates and avoid large differences in mortality by age or calendar year. Because we were mainly interested in the survival by occupation, we also stratified our rates by ISCO-88 1-digit code (79). Net breast cancer survival was computed at 5 years for occupation (ISCO-88 1-digit code), skill level and socio-professional category. We applied a log-rank test to compare the net survival curves between groups.

For the parametric approach, we used the flexrsurv R package (80). We fitted excess hazard model with a cubic spline with 3 knots (1, 5, and 10 years of follow-up) as baseline hazard. Background mortality rates were the same as in the non-parametric survival analysis. Again, we calculated the excess hazards by occupation (ISCO-88 1-digit), skill level and socio-professional category. For each of these variables, we fitted three models. The Model 1 was adjusted for age, calendar period at diagnosis and cancer registry. The Model 2 was furthermore adjusted for nationality and for histological type of tumor, to control for the potential confounding of nationality and smoking. Finally, the Model 3 was additionally adjusted for the tumor stage at diagnosis. We also tested the non-proportional effect of stage using B-Splines (81). In order to compare the fit of our models, we used the Akaike Information Criterion (AIC) (82).

## 3. Results

### 3.1. Cohort description

Of the 20,113 female BC cases diagnosed between 1990 and 2014, we excluded 10,963 cases (55.51%) because of a lack of information on their occupations. Unemployed and job-seeking women (472 cases), who represented 2.34% of the total, were also excluded. The study sample consisted of 8,678 participants (Table 1). Most participants (81%) were Swiss. Sixty-one percent were married, while 19% were single. The mean age at diagnosis was 54.5 ± 9.3 years and the mean duration of follow-up was 9.2 ± 6.3 years. The most represented occupational group was clerks (26%), followed by technicians and associate professionals (24%). About half of the study sample had occupations requiring the second lowest level of skills. In addition, participants were more likely to be in the supervisors/low-level management and skilled labor socio-professional category. Participants in top management and independent occupations accounted for only 3% of study sample.

**Table 1:** Description of the study sample.

Characteristics	*N*	%
Total	8,678	100
Nationality (binary)		
Swiss	7,012	81
Non-Swiss	1,664	19
Unknown	2	0
Civil status		
Single	1,649	19
Married	5,297	61
Widowed	381	4
Divorced	1,351	16
Occupation		
Legislators, senior officials and managers	516	6
Professionals	1,160	13
Technicians and associate professionals	2077	24
Clerks	2,256	26
Service workers and shop and market sales workers	1,509	17
Skilled agriculture and fishery workers	98	1
Craft and related trades workers	269	3
Plant and machine operators and assemblers	92	1
Elementary occupations	701	8
Skill level required for the occupation		
High	1,676	19
Intermediate high	2077	24
Intermediate low	4,224	49
Low	701	8
Socio-professional category		
Top management and independent professions	224	3
Other self-employed	812	9
Professionals and senior management	904	10
Supervisors/low level management and skilled labour	4,926	57
Unskilled employees and workers	1,689	19
In paid employment, not classified elsewhere	123	1
Calendar periods		
1990–1994	1,103	13
1995–1999	1892	22
2000–2004	1,675	19
2005–2009	1876	22
2010–2014	2,132	25
Age at entry (years): mean ± standard deviation	54.5 ± 9.3	
Duration of follow-up (years): mean ± standard deviation	9.2 ± 6.3	

The tumor stage at diagnosis was known for 79% of cases (Table 2). Participants were more likely to be diagnosed at stage I or II (38 and 31% of cases respectively). Ductal carcinoma was the most common histological type of BC (Table 2).

**Table 2:** Characteristics of the breast cancers.

Characteristics	*N*	%
Total	8,678	100
Tumor stage		
Stage I	3,269	38
Stage II	2,655	31
Stage III	662	8
Stage IV	291	3
Unknown	1801	21
Histology type		
Ductal carcinoma	6,487	75
Lobular carcinoma	1,191	14
Other	1,000	12

### 3.2. Ten-year net survival per occupation

**Figure 2:**
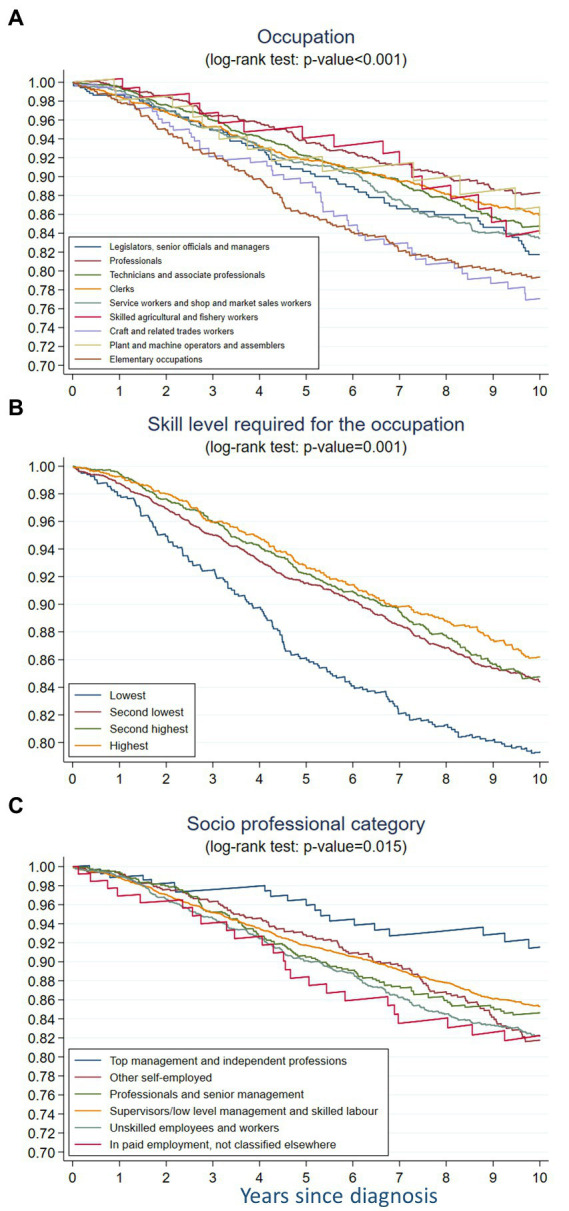
Non-parametric 10-year net survival of breast cancer across **(A)** occupation, **(B)** skill level required for the occupation, and **(C)** socio-professional category among women aged 18–85 in French-speaking Switzerland (1990–2014).

In the nonparametric setting, we observed a strong statistically significant difference in BC survival across occupational groups (*p* < 0.001). Overall, Professionals had the highest survival and Elementary occupations and Craft and related trades workers had the lowest survival, and, with a 10% lower net survival at 10 years compared to Professionals (Figure 2A).

In the parametric analysis, taking elementary occupations as the reference, Craft and related workers had the highest HR in the three models, with 11% higher risk of mortality in the 10 years after BC diagnosis in models 1 and 2 and 23%-risk increase in the model 3 (Table 3). The HR for Plant and machine operators and assemblers was also above 1 but in the Model 3 only. All the other occupational groups had the HR below 1 in the three models, compared to the elementary occupations although the HR were higher in model 3 than in models 1 and 2.

**Table 3:** Hazard ratios (HR) and Confidence Interval (95%-IC) for breast cancer relative survival by work-related variables among women aged 18–85 in French-speaking Switzerland (1990–2014).

Predictor variables	Model 1^a^	Model 2^b^	Model 3^c^
	HR (95%-IC)	HR (95%-IC)	HR (95%-IC)
Occupation			
Legislators, senior officials and managers	0.78 (0.56–1.10)	0.79 (0.56–1.11)	0.92 (0.65–1.30)
Professionals	0.73 (0.56–0.95)	0.73 (0.56–0.96)	0.83 (0.63–1.10)
Technicians and associate professionals	0.81 (0.64–1.01)	0.81 (0.64–1.02)	0.87 (0.68–1.10)
Clerks	0.78 (0.62–0.98)	0.79 (0.63–0.99)	0.81 (0.64–1.02)
Service workers and shop and market sales workers	0.86 (0.68–1.10)	0.87 (0.68–1.10)	0.88 (0.69–1.14)
Skilled agricultural and fishery workers	0.88 (0.49–1.58)	0.89 (0.49–1.60)	0.70 (0.38–1.29)
Craft and related trades workers	1.11 (0.77–1.59)	1.11 (0.77–1.59)	1.23 (0.83–1.81)
Plant and machine operators and assemblers	0.93 (0.50–1.75)	0.94 (0.50–1.76)	1.06 (0.56–2.01)
Elementary occupations	Ref.	Ref.	Ref.
Skill level required for the occupation			
Highest skill level	0.75 (0.58–0.95)	0.76 (0.59–0.97)	0.86 (0.67–1.12)
2nd highest skill level	0.81 (0.64–1.01)	0.82 (0.65–1.04)	0.87 (0.69–1.11)
2nd lowest skill level	0.84 (0.68–1.03)	0.85 (0.69–1.05)	0.86 (0.69–1.07)
Lowest skill level	Ref.	Ref.	Ref.
Socio-professional category			
Top management and independent professions	0.48 (0.26–0.89)	0.49 (0.26–0.90)	0.59 (0.32–1.11)
Other self-employed	1.01 (0.79–1.30)	1.02 (0.79–1.32)	1.00 (0.77–1.29)
Professionals and senior management	1.00 (0.78–1.27)	1.01 (0.79–1.29)	1.06 (0.83–1.37)
Supervisors/low level management and skilled labour	0.86 (0.73–1.01)	0.87 (0.74–1.02)	0.91 (0.77–1.09)
Unskilled employees and workers	Ref.	Ref.	Ref.
In paid employment, not classified elsewhere	0.84 (0.51–1.38)	0.85 (0.52–1.39)	0.87 (0.55–1.39)

a Model 1 is adjusted for age, calendar period and registry.

b Model 2 is adjusted for age, calendar period, registry and nationality and tumor histological type.

c Model 3 is equal to Model 2 additionally adjusted for tumor stage at diagnosis.

### 3.3. Ten-year net survival per skill level

The nonparametric analysis showed a statistically significant difference in BC survival across skill levels required for occupation (*p* < 0.001) (Figure 2B). The survival decreased gradually from that in women with the highest skill level to that in the lowest skill level.

The parametric analysis confirmed this gradient in both Model 1 and Model 2 (Table 3). Having a high skill level had a significant protective effect in both models, but decreased after adjustment for the tumor stage at diagnosis (Model 3). Noteworthy, for women with an intermediate high and an intermediate low skill level, this adjustment induced a very small change.

### 3.4. Ten-year net survival per socio-professional category

In the non-parametric analysis, the BC survival differed across socio-professional categories (*p* = 0.015) (Figure 2C). Women belonging to the top management and independent professions category had the best survival, while women in paid employment not classified elsewhere had the worse one. The second worse BC survival was observed among unskilled employees and workers. The gradient was less obvious then for the skill level variable and the differences between categories were more difficult to disentangle. During the first 2 years of follow-up, the survival among the women in top management and independent professions, those in other self-employed occupations and those working as professionals and senior managers was quite close, then diverged between these three categories and strongly decreased among the latter category. After 3 years of follow-up, the survival of professionals and senior managers became lower than the survival of supervisors/low level managers and skilled laborers, though this observation is based on a small number of cases.

The parametric analysis showed that compared to unskilled employees, women working in top management and independent professions had a twice-lower net rate within 10 years after BC diagnosis without adjusting for the tumor stage (Table 3) and a 40%-lower but statistically non-significant risk after accounting for tumor stage.

## 4. Discussion

### 4.1. Summary of the main results

In this study, we assessed the relationship between the 10-year net survival for breast cancer and occupation, considering three complementary work-related variables: occupation, skill level required for the occupation, and the socio-professional category. Moreover, we applied an original analytical approach including both parametric and non-parametric analyses. To our knowledge, this study is the first to apply such a methodology to investigate the potential work-related determinants of BC survival.

In the non-parametric analysis, we found that the net survival varied across occupations, skill levels, and socio-professional category. Women in elementary occupations, with low skill level, and in paid employment not classified elsewhere, had the lowest BC survival, while professionals, those with the highest skill level and belonging to top management and independent profession category had the highest BC survival. The parametric analysis confirmed this pattern. Considering elementary occupations as reference, all occupations but Craft and related trades had a hazard ratio below 1. Among professionals, technicians and associate professionals, and clerks, the protective effect of occupation (adjusted for age, calendar period and registry) was statistically significant and remained unchanged after further adjusting for nationality and histological type. After adjusting for tumor stage, the hazard ratios remained below 1, though turned statistically non-significant. The same effect was observed in top management and independent professions and in supervisors/low level management and skilled laborers, compared to unskilled workers.

### 4.2. Results interpretation

The results observed in the analysis of the relationship between occupation and skill level are consistent and in line with our research hypothesis. Although the skill-level variable is constructed based on ISCO, it offers a complementary information, helpful for the result interpretation. The skill level was shown as a risk factor in BC incidence, with the highest incidence associated with the highest skill level (12). Such a trend has been also reported for the education (83, 84). However, a recent study using casual mediation analysis concluded that a low educational level is a causal risk factor in the BC development “as it is associated with poor lipid profile, obesity, smoking, and types of physical activity” (85). The protective effect of education with respect to BC survival is also questionable, given the discrepancy between studies (83, 84, 86) and a weak effect-size in the meta-analysis (87).

We observed an increasing survival per increasing skill level required for the occupation, suggesting its protective effect with respect to BC survival. This effect might operate *via* the access to a better (i.e., non-manual, intellectually stimulating and providing a better income) occupation. In such occupations, women have no or low exposure to occupational BC risk factors and benefit from early BC diagnosis and therefore from a less invasive and more effective treatment and return to work. The former was confirmed in our pervious study (12).

The parametric analysis showed clearly that non-manual occupations have a better survival (HR < 0.85), with technicians and associate professionals at a cross-section between manual and non-manual occupations. Professionals and clerks had the lowest HRs in all models, compared to women working in elementary occupations, used as reference. It worth mentioning that in the previous analysis, elementary occupations had a significantly reduced risk of BC, but a higher proportion of advanced (III or IV) and missing tumor stage at diagnosis (12). In Switzerland, elementary occupations have the highest proportion (>60%) of part-time workers, and women represent three fourths of them (88). Part-time work could be either protective or a risk factor for survival depending on whether it is voluntary or involuntary, for example when it is not possible to return to full-time work (59). Nevertheless, the evidence is still very scarce. Some women with low skill levels belong to this occupational group, and the result could be explained, at least partially, by financial constrains (60, 89).

The results of analysis according to the socio-professional category are less easily interpretable. On the one hand, the protective effect of the top management and independent professions in both parametric and non-parametric analysis appears a consistent finding overall. On the other hand, low level management corresponded to a better survival and hazard ratio than the reference category (unskilled workers). Furthermore, professional and senior management and other self-employment exhibited a hazard ratios similar to the reference category and higher than the low level management. However, net survival during the follow-up showed different patterns between these groups which may reveal non proportional effect of occupation. The classification of socio-professional categories used in this study accounts for all levels of the population’s socio-professional structure (90). Therefore, the risk of misclassification can reasonably be ruled out. A tentative explanation of this finding could thus lie in a set of conditions associated with the above-mentioned socio-professional categories, especially when considered the temporal change in survival in the non-parametric analysis. This could be the BC treatment-related side effects, and particularly, their impact on mental health and cognitive functions (52, 91–96). In fact, the return to work in these socio-professional groups can be easier than elsewhere, but long-term or chronic functional cognitive deficiencies can impact their work performance and participation (52). Cognitive difficulties affect approximatively 30% of BC survivors (97, 98) and can be more troublesome than physical side effects (52, 99).

### 4.3. Methodological aspects

Despite an intensive effort to complete and confirm this data using cancer registry records, the tumor stage at diagnosis was missing for 21% of women in our study sample. To properly manage missing values for this important variable, we used multiple imputations. The complete case analyses provided estimated hazard ratios with wider confidence intervals (Supplementary Table S1). This suggests that the imputation permitted to increase the precision in study results.

Regarding the occupation variable, the 2-digit ISCO variable showed the best predictive accuracy for all work-related health measures (63). However, the AIC was smaller in the model with the occupation variable coded using one-digit ISCO-88 than the model with occupation coded using two-digit ISCO-88 codes, suggesting that the former fitted the data better than the latter. Therefore, we used the occupation variable coded using one-digit ISCO-88 in our analyses. We recognize that occupational groups at this large level of aggregation (1 or 2 digits) might be insufficiently specific to incorporate factors directly related to occupational exposures and working conditions. Information on working conditions before and after diagnosis along with information on RTW after cancer treatment were not available but are important to collect and analyze with respect to cancer survival in the future. RTW reduces economic hardship, maintains mental well-being, facilitates physical recovery, and may improve BC survival (47, 100). Consequently, we believe that analyses at a finer level of ISCO-88 (3 or 4 digits), combined with information on the duration of sick leave, RTW, and working conditions and exposures could improve the understanding of net BC survival.

### 4.4. Strengths and limitations

The use of the international classification of occupation and skill level required for the occupation is a major strength of this study. Standardized classifications enable a common and replicable definition and measurement of these work-related variables. A high completeness of case ascertainment in western Swiss cancer registries during the study period along with the incidence information of a very good quality (101) is another strength of this study. By focusing on female BC we addressed the recommendation of the European Union information agency for occupational safety and health to investigate female workers’ health more specifically (102).

From the methodological point of view, this study has also brought several advantages. The application of the non-parametric method enabled us calculating the BC net survival for each of the three occupational variables without making any specific assumption. Furthermore, the application of the parametric method enabled us quantifying the differences between groups in terms of hazard ratios and test the proportional hazards assumption for the tumor stage at diagnosis.

The use of a relative survival framework in our study was appropriate to investigate inequalities in BC survival. This permitted accounting for disparities in mortality between study groups with respect to multiple causes of death (15). This was done using life table that we stratified by occupation. As a result, the socio-economic conditions were more homogenous within each strata. This is important to emphasize because socio-economic conditions are expected to influence general mortality (79). This enabled us limiting biases in the estimate of the effect of this variable but also, to a lesser extent, in the estimates of the effects of the other covariates included in the model (103). Because of the association between mortality and occupational variables, we believe that future studies on occupational factors should also focus on relative survival methods using life table stratified by occupation.

Regarding limitations, occupation was missing for 55% of women in the SNC, and the study sample was half smaller after exclusion of these cases. A comparison of participants with and without occupational information showed that they had similar net survival characteristics (Supplementary Table S2). However, we could not fully rule out a selection bias since no comparison of the distribution of work-related variables for patients with and without information on occupation was possible.

Assigning occupations as a time-dependent variable based on two time points could result in some misclassification, especially given that cancer survivors have a less stable employment trajectory than other workers (59). Nevertheless, the information on the occupation at the time of the federal censuses was correct and we believe that it was assigned accurately, as the majority of patients kept the same occupation between the two censuses (104, 105). Having occupational information at the time of diagnosis would be better, but it was not available.

The quality of occupational data in all western Swiss cancer registries and in the SNC were assessed in a prior study and varied across registries (106). Thus, to avoid differential misclassification of occupations, we decided to use SNC data rather than registry data. Moreover, we were constrained to aggregating the occupation under one-digit codes, reducing the variability of occupational situations, due to the limited number of observations per occupational group. This can explain a likely lack of statistical power in parametric analysis and non-statically significant hazard ratios.

Finally, information on smoking, duration of sick leave, and working conditions after RTW, particularly the night shift work, was not available to study their effect on BC survival. Information on BC treatment was also unavailable. Yet, since the implementation of the Swiss federal law on cancer registration in 2020, all Swiss cancer registries should collect this information systematically (107). The use of these data in future large, nationwide, or international studies will allow a more accurate estimation of factors affecting net BC survival. Such studies are important to informing health and social protection systems, which should guarantee appropriate work conditions for cancer survivors and educate them on their rights and obligations during sick leave (108). It is essential that clinicians and institutions consider work-related issues in BC patients and perform adequate organizational and normative interventions, particularly in the most vulnerable occupational groups (38).

## 5. Conclusion

This study reports the net survival for breast cancer across three complementary occupation-related variables: occupation, skill level required for the occupation, and the socio-professional category of employment. We found that the net BC survival depended on these variables. The lowest skill level was associated with the worst survival prospects, while working in non-manual occupations in general, and in top management and independent professions particularly was related to a better survival. In the parametric models, the adjustment for the histological type of BC and tumor stage at diagnosis allowed us to control for the effect of these variables and indirectly control for smoking. After this adjustment, the hazard ratio estimates are closer to unity with few impacts on their order suggesting a limited impact of the inequality in diagnosis on the net survival.

As this study was conducted using a limited set of covariates and a relatively small sample, further studies are required, taking into account smoking habits and treatments administrated to the BC patients. Information on RTW and working conditions before and after BC diagnosis will also be highly valuable to analyzing their effect on BC net survival in large nationwide or international studies. Such studies are essential to informing health and social protection systems, which should guarantee appropriate work conditions for BC survivors, beneficial for their quality of life and survival.

## Data availability statement

The datasets presented in this article are not readily available because The data were generated under the mandate of the Swiss federal statistical office cannot be used for any other study. Requests to access the datasets should be directed to irina.guseva-canu@unisante.ch.

## Ethics statement

The studies involving human participants were reviewed and approved by Cantonal Ethics Committees of Bern and Zurich and by the Commission cantonale d’éthique de la recherche sur l’être humain (CER-VD) (Project-ID 2018–02077). Written informed consent for participation was not required for this study in accordance with the national legislation and the institutional requirements.

## Author contributions

NB, MG, and IC: conceptualization. NB: data curation. IC: funding acquisition. PA, J-LB, EF, SG, IK, MM, and ER: resources. NB, MG, and IC: validation. NB and IC: writing—original draft. NB, MG, PA, J-LB, EF, SG, IK, MM, ER, and IC: writing—review and editing. All authors contributed to the article and approved the submitted version.

## Funding

This research was funded by the Swiss Cancer Research Foundation and the Swiss Cancer League, grant number No KFS-4699-02-2019. Open access funding provided by the University of Lausanne.

## Conflict of interest

The authors declare that the research was conducted in the absence of any commercial or financial relationships that could be construed as a potential conflict of interest.

## Publisher’s note

All claims expressed in this article are solely those of the authors and do not necessarily represent those of their affiliated organizations, or those of the publisher, the editors and the reviewers. Any product that may be evaluated in this article, or claim that may be made by its manufacturer, is not guaranteed or endorsed by the publisher.
