# Supplementary material for: Breast cancer and occupation: Non-parametric and parametric net survival analyses among Swiss women (1990–2014)
Source: Front Public Health. 2023 Apr 5;11:1129708. doi: 10.3389/fpubh.2023.1129708 (PMC10115164; doi:10.3389/fpubh.2023.1129708)
Supplement: Supplementary file 1 [file Table_1.DOCX]

Supplementary material table S1. Hazard ratios (HR) and Confidence Interval (95%-IC) for breast cancer relative survival by work-related variables among women aged 18-85 in French-speaking Switzerland (1990-2014) with complete data on tumor stage at diagnosis

| **Predictor variables** |  | **HR (95%-CI)** | |  |
| --- | --- | --- | --- | --- |
| Occupation |  |  |  |  |
| Legislators, senior officials and managers |  | 0.56 | (0.30-1.05) |  |
| Professionals |  | 0.56 | (0.34-0.91) |  |
| Technicians and associate professionals |  | 0.65 | (0.42-0.99) |  |
| Clerks |  | 0.52 | (0.34-0.82) |  |
| Service workers and shop and market sales workers |  | 0.59 | (0.38-0.90) |  |
| Skilled agricultural and fishery workers |  | 0.34 | (0.06-1.76) |  |
| Craft and related trades workers |  | 0.89 | (0.48-1.63) |  |
| Plant and machine operators and assemblers |  | 0.04 | (0.00-104) |  |
| Elementary occupations |  | Ref. |  |  |
| Skill level required for the occupation |  |  |  |  |
| Highest skill level |  | 0.56 | (0.35-0.89) |  |
| 2nd highest skill level |  | 0.65 | (0.43-0.99) |  |
| 2nd lowest skill level |  | 0.57 | (0.38-0.84) |  |
| Lowest skill level |  | Ref. |  |  |
| Socio-professional category |  |  |  |  |
| Top management and independent professions |  | 0.61 | (0.22-1.72) |  |
| Other self-employed |  | 1.32 | (0.86-2.01) |  |
| Professionals and senior management |  | 1.27 | (0.85-1.92) |  |
| Supervisors/low level management and skilled labour |  | 0.99 | (0.73-1.36) |  |
| Unskilled employees and workers |  | Ref. |  |  |
| In paid employment, not classified elsewhere |  | 1.44 | (0.44-4.65) |  |

Models are adjusted for age, calendar period, registry and nationality, tumor histological type, and tumor stage at diagnosis.
