# Supplementary material for: Breast cancer and occupation: Non-parametric and parametric net survival analyses among Swiss women (1990–2014)
Source: Front Public Health. 2023 Apr 5;11:1129708. doi: 10.3389/fpubh.2023.1129708 (PMC10115164; doi:10.3389/fpubh.2023.1129708)
Supplement: Supplementary file 2 [file Table_2.docx]

Supplementary material table S2. Comparison of the non-parametric net survival estimated in the study sample (all women) and the sub-sample of women with available occupational data

|  | **All women** |  |  |  | **Women with avalaible occupation only** | | |
| --- | --- | --- | --- | --- | --- | --- | --- |
|  | Net survival | [95% Conf. Int.] | |  | Net survival | [95% Conf. Int.] | |
| 5-year | 0.91 | 0.90 | 0.91 |  | 0.92 | 0.91 | 0.92 |
| 10-year | 0.84 | 0.83 | 0.84 |  | 0.85 | 0.84 | 0.86 |
| Stage |  |  |  |  |  |  |  |
| I |  |  |  |  |  |  |  |
| 5 | 0.99 | 0.99 | 1.00 |  | 0.99 | 0.99 | 1.00 |
| 10 | 0.97 | 0.96 | 0.98 |  | 0.98 | 0.96 | 0.99 |
| II |  |  |  |  |  |  |  |
| 5 | 0.94 | 0.93 | 0.95 |  | 0.94 | 0.93 | 0.95 |
| 10 | 0.86 | 0.84 | 0.87 |  | 0.87 | 0.85 | 0.88 |
| III-VI |  |  |  |  |  |  |  |
| 5 | 0.63 | 0.61 | 0.65 |  | 0.68 | 0.65 | 0.72 |
| 10 | 0.47 | 0.45 | 0.50 |  | 0.52 | 0.48 | 0.56 |
| Before 2000 |  |  |  |  |  |  |  |
| 5-year | 0.88 | 0.87 | 0.89 |  | 0.88 | 0.87 | 0.89 |
| 10-year | 0.80 | 0.79 | 0.81 |  | 0.80 | 0.78 | 0.82 |
| After 2000 |  |  |  |  |  |  |  |
| 5-year | 0.92 | 0.91 | 0.93 |  | 0.94 | 0.93 | 0.95 |
| 10-year | 0.86 | 0.85 | 0.87 |  | 0.89 | 0.88 | 0.90 |
